# Development of a rapid neutralization testing system for Rhinovirus C15 based on the enzyme-linked immunospot assay

**DOI:** 10.3389/fmicb.2022.983656

**Published:** 2022-09-23

**Authors:** Zhenhong Zhou, Rui Zhu, Hongwei Yang, Longfa Xu, Hao Chen, Yuanyuan Wu, Zhichao Yin, Qiongzi Huang, Dongqing Zhang, Che Liu, Yuqiong Que, Jun Zhang, Ningshao Xia, Tong Cheng

**Affiliations:** State Key Laboratory of Molecular Vaccinology and Molecular Diagnostics, School of Life Sciences, School of Public Health, National Institute of Diagnostics and Vaccine Development in Infectious Diseases, Xiamen University, Xiamen, China

**Keywords:** Rhinovirus C15, neutralization assay, enzyme-linked immunospot assay, neutralizing antibody, seroprevalence

## Abstract

Human Rhinoviruses (RVs) are dominant pathogens causing a wide range of respiratory tract diseases, posing a huge threat to public health worldwide. Viruses belonging to the RV-C species are more likely to cause severe illnesses and are strongly associated with asthma onset or exacerbations than RV-A or RV-B. Rapid and sensitive detection of neutralizing antibodies (NAbs) against RV-C can promote the development of vaccines and antiviral drugs and help in the diagnosis of viral infection. In this study, a rapid neutralization testing system for RV-C15, based on an enzyme-linked immunospot assay (Nt-ELISPOT) was developed. A monoclonal antibody (MAb), named 9F9, with high binding efficacy for RV-C15 conjugated to horseradish peroxidase (HRP), was used to detect RV-C15-infected cells at a concentration of 2 μg/ml. The optimal infectious dose of RV-C15 was set at 1 × 10^4^ TCID_50_/well and the cells were fixed with 0.5% formaldehyde diluted in PBS after incubation for 20 h. Compared with the traditional cytopathic effect (CPE)-based neutralization assay (Nt-CPE), Nt-ELISPOT significantly shortened the detection period and showed good consistency with the detection of neutralizing titers of both sera and NAbs. Using Nt-ELISPOT, three anti-RV-C15 NAbs were obtained with IC_50_ values of 0.16, 0.27, and 11.8 μg/ml, respectively. Moreover, 64 human serum samples collected from a wide range of age groups were tested for NAb against RV-C15 by Nt-ELISPOT. The total seroprevalence was 48.4% (31/64) and the positive rate was lowest in the group under 6 years old. Thus, the Nt-ELISPOT established in this study can be used as a high-throughput and rapid neutralization assay for the screening of NAbs and for seroepidemiological investigation against RV-C15.

## Introduction

Human Rhinoviruses (RVs), a group of non-enveloped, single-stranded RNA viruses belonging to the genus *Enterovirus* of the family *Picornaviridae*, are the dominant pathogens that cause respiratory illnesses (Jacobs et al., 2013). RV infections occur worldwide and nearly year-round and contagious easily *via* contact (either direct or through a fomite) or aerosol, inflicting all age groups. RV infections can cause more than half of the upper respiratory tract infections (URTIs; Jacobs et al., 2013), generally known as the common cold, and have been associated with wheezing (Jartti et al., 2004; Liu et al., 2017), bronchiolitis (Hasegawa et al., 2019), pneumonia (Imakita et al., 2000), and acute otitis media (Chantzi et al., 2006; Seppälä et al., 2020). Importantly, they have also been associated with the exacerbation of chronic respiratory illnesses such as asthma (Ortega et al., 2021) and chronic obstructive pulmonary disease (COPD; Cafferkey et al., 2020). To date, more than 160 RV serotypes have been identified and classified into three species (RV-A, RV-B, and RV-C; Jacobs et al., 2013; Bochkov and Gern, 2016). Since RV-C species were first discovered in 2006 (Arden et al., 2006; Bochkov et al., 2011), a growing number of studies have shown that RV-C can cause more severe illnesses in infants and children than RV-A or RV-B, including those requiring hospitalization, and is more strongly associated with asthma onset or exacerbations (Bizzintino et al., 2011; Drysdale et al., 2014; Su et al., 2020; Esneau et al., 2022). The resulting substantial public health threat and ongoing disease burden underscores the necessity and urgency of developing effective treatments for RV infections, particularly RV-C.

The humoral immune response plays a crucial role in combating and protecting against pathogen invasion, and is the underlying protective mechanism of most successful vaccines. Since neutralizing antibody (NAb) levels are generally considered a dominant indicator of protective immunity, monitoring the seroprevalence against RV infection in susceptible populations is of great significance for understanding the history of herd immunity and previous infection (Choi et al., 2021; Esneau et al., 2022). Moreover, the discovery and characterization of highly potent NAbs have contributed to the development of vaccines and antiviral drugs (Walker and Burton, 2018; Graham et al., 2019; Touabi et al., 2021). However, NAb responses to RV-C have been poorly analyzed, and no NAb response against RV-C has yet been described. The traditional cytopathic effect (CPE)-based neutralization assay (Nt-CPE) is a commonly used neutralization method for Rhinoviruses, but it is not suitable for the detection of a large number of samples because it is time-consuming and labor-intensive. Therefore, a rapid and efficient neutralization assay needs to be developed.

**Figure 1:**
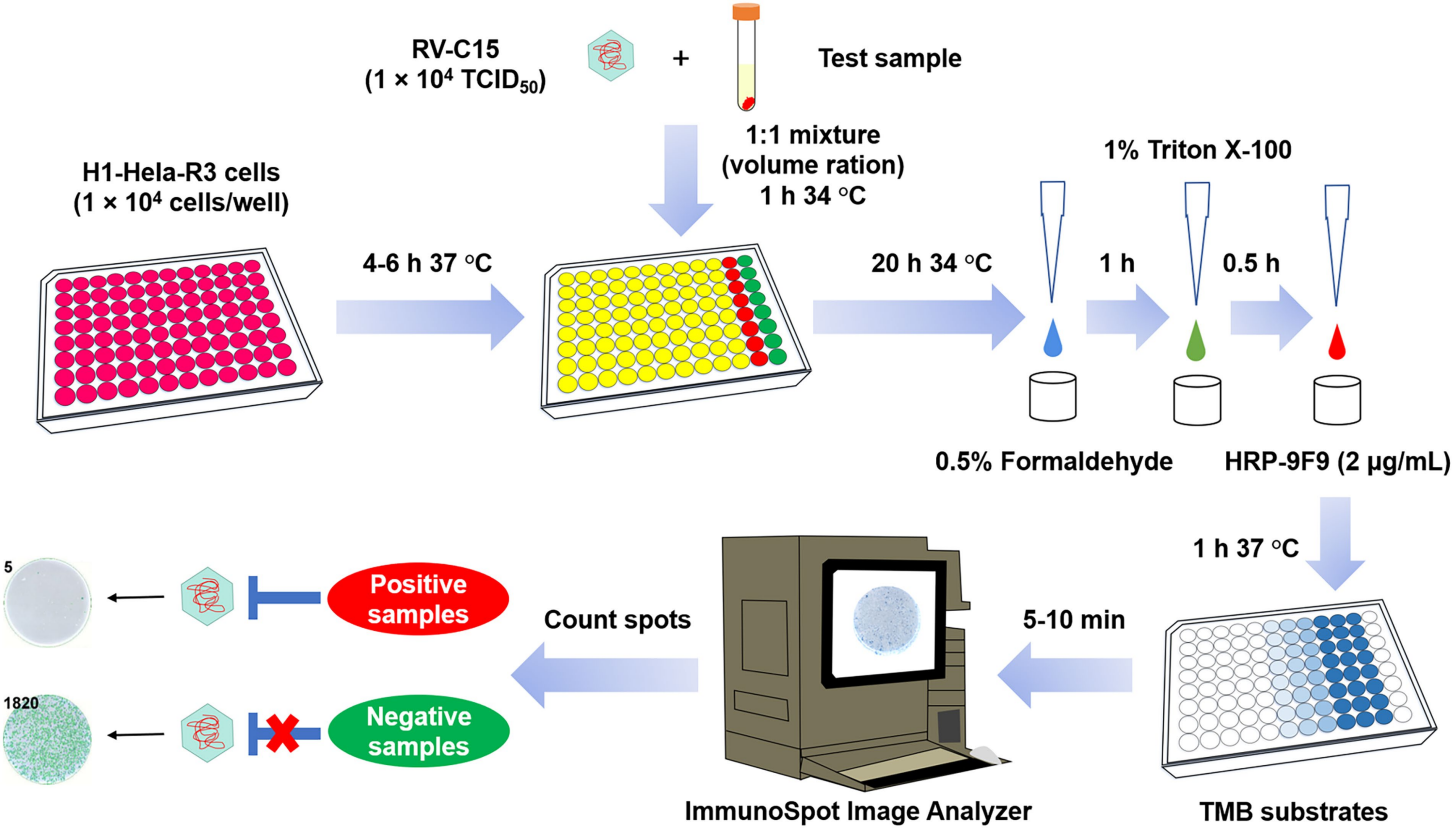
Flow diagram of the Nt-ELISPOT for RV-C15. First, H1-Hela-R3 (1 × 10^4^ cells/well) were seeded into 96-well plates at least 4 h prior to infection. Then, the serial dilutions of the test samples were mixed with an equal volume of RV-C15 (1 × 10^4^ TCID_50_) and incubated at 34°C for 1 h. The mixtures were then added to the cell plates, with the virus control wells and the cell control wells included on the last two columns of each plate. After incubation at 34°C for 20 h, cells were fixed with 0.5% formaldehyde and permeabilized with 1% Triton X-100, and then incubated with HRP-9F9 (2 μg/ml) at 37°C for 1 h. Following at least five washes with PBST (0.05% Tween 20 in PBS), the infected wells were visualized as blue spots after adding the TMB substrates. Spots were counted using an ImmunoSpot@S5 UV Analyzer. The neutralizing titer of the test sample was defined as the highest dilution that completely inhibited more than 50% of the spots.

In 2021, a novel quantitative PCR-based assay for detecting NAbs against several RV serotypes was reported by Choi et al. However, this method is relatively complex and inefficient, and can only be conducted in 24-well cell culture plates, requiring the extraction of viral nucleotides and RT-PCR after 3 days of incubation (Choi et al., 2021). Recently, an ELISPOT-based neutralization assay (Nt-ELISPOT) has been developed and it is widely used for the detection of NAb levels against many types of viruses, such as SARS-CoV-2 (Park et al., 2021), CVB1 (Wu et al., 2022), CVA10 (Liu et al., 2019), ZIKA (Li et al., 2020), and HSV-1 (Luo et al., 2016). The established Nt-ELISPOT showed characteristics such as high sensitivity, objectivity, efficiency, and is high throughput. In this study, the prevalent RV-C15 serotype was selected to study and whether Nt-ELISPOT could be optimized and applied to RV was also tested. Finally, a rapid and efficient Nt-ELISPOT against RV-C15 was successfully developed and used for the detection of NAb levels in a cohort and screening of neutralizing monoclonal antibodies (MAbs). Here, we described a detailed protocol of Nt-ELISPOT to evaluate and measure NAb against RV-C15 (Figure 1). This assay allowed the neutralization test to be completed within 30 h in a 96-well plate format.

## Materials and equipment

Table 1 lists the key materials and equipment used in this study. Most reagents required for this system are commercially available. Additional reagents and serum samples are listed in the Supplementary material.

**Table 1:** Key resource.

Reagent or resource	Source	Identifier
*Antibodies*		
Anti-RV-C15 mouse monoclonal antibody 9F9	This study	N/A
HRP-conjugated Goat anti-Mouse IgG	This study	N/A
Alexa Fluor^®^ 488 goat anti-mouse IgG (H + L)	Invitrogen	Cat# A-11001
*Bacterial and virus strains*		
RV-C15 W10 strain	This study	GenBank: GU219984
RV-A2 HGP strain	ATCC	GenBank: AB079139
RV-B14 1,059 strain	ATCC	GenBank: EU870450
DH5α competent cell	TianGen	Cat# CB101
*Chemicals, peptides, and recombinant proteins*		
Dulbecco’s modified Eagle’s medium	Sigma-Aldrich	Cat# D6429
RPMI-1640 medium	Thermo Fisher Scientific	Cat# 11875–093
Fetal bovine serum (FBS)	Thermo Fisher Scientific	Cat# 10099141
Penicillin–Streptomycin	Thermo Fisher Scientific	Cat# 15140–122
l-Glutamine	Thermo Fisher Scientific	Cat# 25030–081
Hypoxanthine	Sigma-Aldrich	Cat# H9377
Methotrexate	Sigma-Aldrich	Cat# PHR1396
Thymidine	Sigma-Aldrich	Cat# T9250
Lipofectamine 3000	Thermo Fisher Scientific	Cat# L3000015
Puromycin	InvivoGen	Cat# ant-pr-1
Aluminum adjuvant	This study	N/A
Phosphate-buffered saline (PBS)	Thermo Fisher Scientific	Cat# 10010031
TMB substrate	Wantai BioPharm, Beijing, China	N/A
Gelatin	Sigma-Aldrich	Cat# G7765
Casein	Sigma-Aldrich	Cat# C8654
Sulfuric acid (H_2_SO_4_)	Wantai BioPharm, Beijing, China	N/A
Paraformaldehyde	Merck	Cat# 16005
Bovine serum albumin (BSA)	Sigma-Aldrich	Cat# A1933-100G
Triton X-100	AMRESCO	Cat# 0694
DAPI	Merck	Cat# D9542
Formaldehyde	Sigma-Aldrich	Cat# F8775
EZ-Link Maleimide activated Horseradish Peroxidase (HRP)	Thermo Fisher Scientific	Cat# 31485
*Critical commercial assays*		
MEGAscript T7 transcription kit	Thermo Fisher Scientific	Cat# AM1334
AT Protein A Diamond	Bestchrom	Cat# AA0273
*Experimental models: cell lines*		
H1-Hela	ATCC	Cat# CRL-1958
HEK293T/17	ATCC	Cat# CRL-11268
H1-Hela-R3	This study	N/A
Myeloma cells (Sp2/0)	This study	N/A
*Experimental models: organisms/strains*		
BALB/c mice	Slac Laboratiry Animal Co., Shanghai, China	N/A
*Recombinant DNA*		
pLV-FLAG-CDHR3Y529	This study	N/A
psPAX2	Addgene	Cat# 12260
pMD2.G	Addgene	Cat# 12259
pMDA2-RV-C15 infectious cDNA clone	This study	N/A
*Software and algorithms*		
GraphPad Prism 8.0	GraphPad Software	https://www.graphpad.com/scientific-software/prism/
VassarStats website	N/A	http://vassarstats.net/index.html
ImmunoSpot analysis software 5.0	Cellular Technology Ltd., USA	N/A
*Other*		
100-mm cell culture dish	NEST	Cat# TCD-100
Flat bottom 96-well plate microplate	LabServ	Cat# 310109008
U-shaped 96-well plate microplate	Yijiamei Experimentation Equipment Co., Xiamen, China	N/A
Opera Phenix confocal microscope	Perkin Elmer	N/A
ImmunoSpot@S5 UV Analyzer	Cellular Technology Ltd., USA	N/A

## Methods

In this study, we explored and optimized several parameters of experimental system, including the detection antibody, cell-fixed solution, infectious dose, and incubation time (see Results 1–3 for details), and established a rapid and efficient neutralizing testing system for RV-C15 based on ELISPOT. Here, we provided a step-by-step procedure of the established Nt-ELISPOT protocol for testing NAb titers of samples (sera, MAbs, or hybridoma cells’ culture supernatants) under BSL-2 conditions. Additional experimental methods are provided in the Supplementary material.

### Step-by-step method details of Nt-ELISPOT

#### Seed the plates for Nt-ELISPOT (4–6 h before)

This section aims to harvest cells and plate them for neutralization test.

- Resuspend H1-Hela-R3 cells thoroughly with a serological pipette.
- Take cell solution for cell counting by automated cell counting instrument.
- Dilute cell solution to 1 × 10^5^ cells/ml.
- Prepare 11 ml of H1-Hela-R3 cells in DMEM (supplemented with 2% FBS, 100 μg/ml streptomycin, 100 U/ml penicillin, and 2 mM l-glutamine) for each flat-bottom 96-well plate as required.
- Use a multiple-channel pipette to evenly distribute 100 μl diluted cell solution to flat-bottom 96-well plates, resulting in 1 × 10^4^ cells per well.
- Place the cell plates into the incubator at 37°C with 5% CO_2_ and incubate for 4–6 h.

#### Prepare the dilution plates

**Figure 2:**
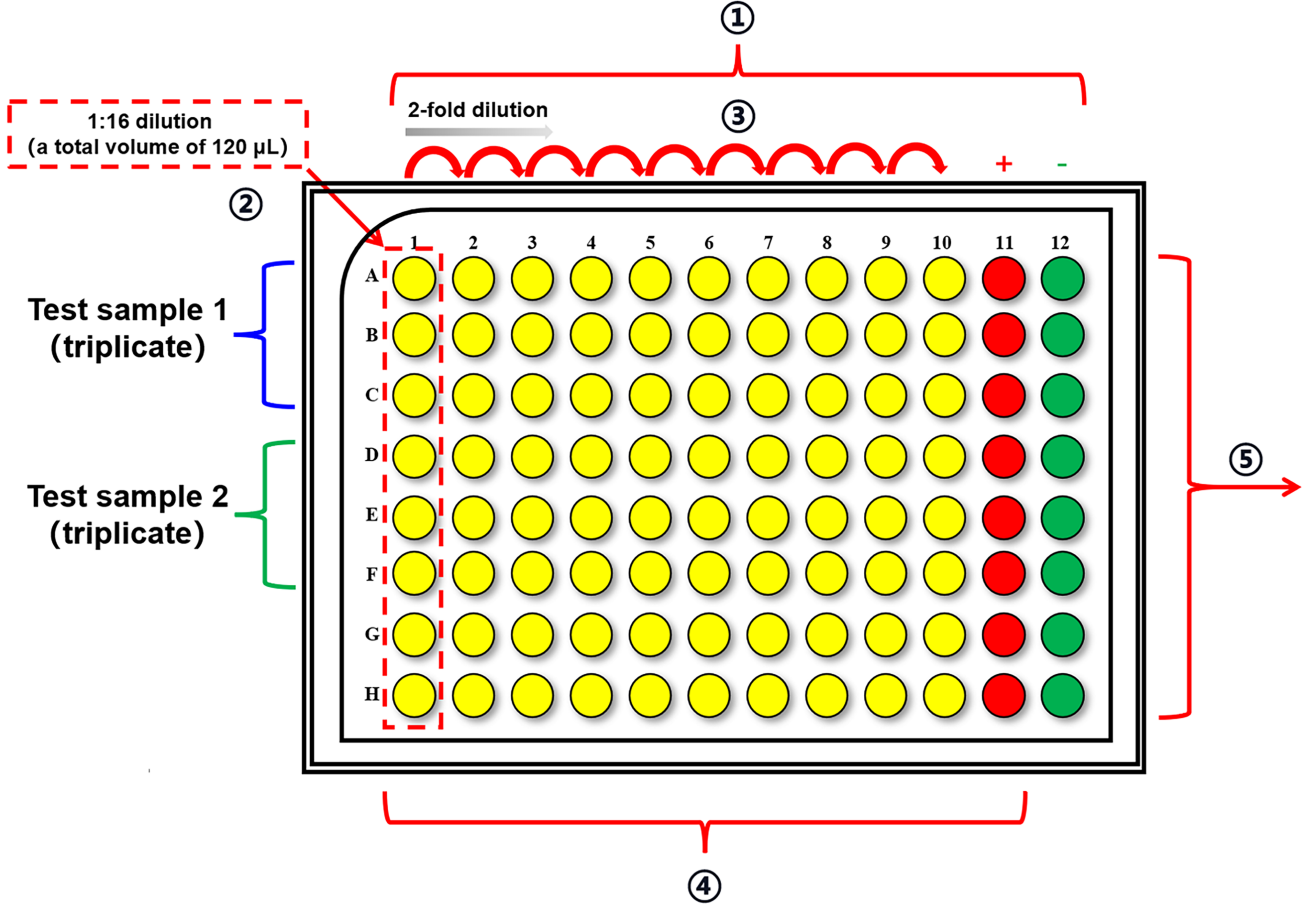
The layout of U-shaped 96-well plates for incubation of samples and viruses. ① Add 112.5 μl serum-free DMEM to column 1 and 60 μl serum-free DMEM to columns 2–12 in U-shaped 96-well plates. ② Add 7.5 μl of test samples to each well in column 1 and each sample is in triplicate. ③ Perform a 2-fold serial dilution in columns 1–10. ④ Add 60 μl of diluted virus stock to columns 1–11 and incubate the plates at 34°C for 1 h. Column 11 is employed as the virus control wells. Add additional 60 μl serum-free DMEM to column 12 to serve as cell control wells. ⑤ Transfer the mixtures to the pre-seeded cell plates and incubate the plates at 34°C for 20 h.

Note: Once the above steps are completed, proceed to the next step or keep at 4°C.

This section aims to prepare the dilution plate as described in Figure 2, which is carried out in U-shaped 96-well plates.

- In the column 1, prepare the starting dilution for test sample in serum-free DMEM, adjusted to a final volume of 120 μl, and then mix by pipetting at least 15 times. For example, add 112.5 μl serum-free DMEM to each well, followed by 7.5 μl of test samples to each well.
  Note: Each sample should be set at least in duplicate. All serum samples should be inactivated at 56°C for 30 min prior to testing.
- In the remaining wells, add 60 μl of serum-free DMEM.
- Perform a 2-fold serial dilution in columns 1–10: Using an eight-channel pipette, take 60 μl of supernatant from the column 1 and pipette into column 2 and mix by pipetting at least 15 times. Repeat for column 2 to column 3, remembering to change tips in between, until column 10.

#### Infection

This section aims to fully incubate the diluted samples with viruses, and transfer the mixtures onto the pre-seeded cell plates.

Note: All subsequent steps should be performed under BSL-2 conditions.

- Dilute RV-C15 virus stock to 1 × 10^4^ TCID_50_/60 μl in serum-free DMEM.
- Prepare 6 ml of RV-C15 (1 × 10^4^ TCID_50_/60 μl) for each plate as required.
- Use a multiple-channel pipette to evenly distribute 60 μl diluted virus stock to each well of columns 1–10 containing 2-fold serial dilutions of test samples and column 11 in the U-shaped 96-well plates and mix by pipetting at least five times.
- Add additional 60 μl serum-free DMEM to column 12 to serve as cell control wells.
- Incubate the U-shaped 96-well plates at 34°C for 1 h.
- After the incubation step, use a multiple-channel pipette to transfer the mixtures of U-shaped 96-well plates to the pre-seeded cell plates (96-well), remembering to change tips with every transfer.
- Place the cell plates back into the incubator at 34°C with 5% CO_2_ and incubate for 20 h.

Note: The infectious dose of virus and incubation time should be explored and optimized. Each plate should include a cell control and a virus control and each sample should be set at least in duplicate.

#### ELISPOT assay

This section aims to visualize each virus-infected cell as a spot after an enzyme-catalyzed color reaction, using an ELISPOT assay. The high-affinity MAb 9F9 against RV-C15 was selected as the detection antibody in this study, and the optimal concentration of HRP-9F9 was determined by optimization. The compositions and storage conditions of buffers and solutions used in this assay are described in Table 2.

**Table 2:** Compositions and storage conditions of buffers and solutions used in Nt-ELISPOT.

Name	Recipe	Storage
Fixation solution	0.5% formaldehyde in PBS	Store at 4°C, good for 3 months
Permeabilization solution	1% Triton X-100 in PBS	Store at 4°C, good for 3 months
ED buffer	2% gelatin and 5% casein in PBS	Store at 4°C, good for 1 month
Antibody solution	2 μg/ml HRP-conjugated 9F9 in antibody buffer	Freshly made
Washing buffer (PBST)	0.05% Tween 20 in PBS	Store at r.t., good for 2 months
Staining solution	1:1 mixture of TMB substrate A and B + 250 mM dextran and 2 mM Sulfurized dextran	Freshly made

r.t., room temperature ranging from 20°C to 25°C.

Note: All subsequent steps should be performed under BSL-2 conditions. Add all buffer and solution slowly and gently to the walls of plate wells to prevent resuspending the cells. No need to change tips when adding or removing buffer or solution, except in Step a.

- At 20 h post-infection, remove the cell plates from the incubator, and then remove the overlay medium from the plates by a vacuum aspirator or a multiple-channel pipette, remembering to change tips.
- Using a multiple-channel pipette, add 100 μl of fixation solution to each well, and incubate the plate at room temperature (20–25°C) for 1 h.
  Note: Fixation solution should be explored and optimized. After fixing the cells, proceed to the next step or keep at 4°C for no more than 1 day.
- Remove the overlay fixation solution from the plates by a vacuum aspirator or a multiple-channel pipette.
- Using a multiple-channel pipette, add 100 μl of permeabilization solution to each well, and then incubate the plates at room temperature (20–25°C) for 30 min.
- Remove the overlay permeabilization solution from the plates by a vacuum aspirator or a multiple-channel pipette.
- Wash the cells in all wells once with 300 μl PBS buffer.
  Note: After washing, ensure complete removal of any residual liquid.
- Dilute the detection antibody HRP-9F9 to 2 μg/ml in ED buffer, and add 100 μl antibody solution to each well by a multiple-channel pipette, and then incubate at 37°C for 1 h.
  Note: HRP-9F9 should be stored at −20°C in the dark until ready to use.
- After incubation, remove the overlay solution from the plates by a vacuum aspirator or a multiple-channel pipette.
- Wash the cells in all wells at least five times with 300 μl washing buffer and incubate for 5 min for each wash at room temperature.
- After the last wash, tap or shake the plates dry to remove the excess of the washing buffer.
- Prepare staining solution under dark conditions, and add 100 μl staining solution to each well by a multiple-channel pipette, and then incubate at room temperature for 10 min.
- After incubation, remove the overlay solution from the plates by a vacuum aspirator or a multiple-channel pipette, and then tap or shake the plates dry to remove the excess of the staining solution.

#### Scanning the plates and counting the spots

- Scan the plates with an ImmunoSpot@S5 UV Analyzer using the blue color system, and count the spots with ImmunoSpot professional analysis software (version 5.0).
- The counting parameters are set as follows: the spot size is set between 0.0001 (Min) and 9.6296 (Max) mm^2^, the sensitivity is set to an arbitrary value between 180 and 200, and the background balance and the diffuse spot process is 0.

#### Neutralizing titer calculation

- The inhibition rate of each sample could be calculated using the following equation:

$$\begin{array}{c} P\left(\%\right)=[1-\left(N_{\mathrm{test}}-N_{\mathrm{cell control}}\right)\\ \;/\left(N_{\mathrm{virus control}}-N_{\mathrm{cell control}}\right)\times 100\% \end{array}$$

Note: where *P* is the inhibition rate of the sample, and *N*_test_, *N*_cell control_, and *N*_virus control_ are the average number of spots in the test wells, cell control wells, and virus control wells, respectively.
The neutralizing titer of each sample could be defined as the reciprocal of the highest dilution that completely inhibited more than 50% of the spots. The neutralizing titer of serum sample with values ≥16 is considered the threshold for positivity (Liu et al., 2011, 2019; Zhu et al., 2018). *P* (%) should be plotted against each dilution using four-parameter logistic curve, and 50% inhibitory concentration (IC_50_) value of each sample could be calculated by nonlinear dose–response regression analysis using GraphPad Prism software.

## Results

### Characterization of the detection antibody in the Nt-ELISPOT

**Figure 3:**
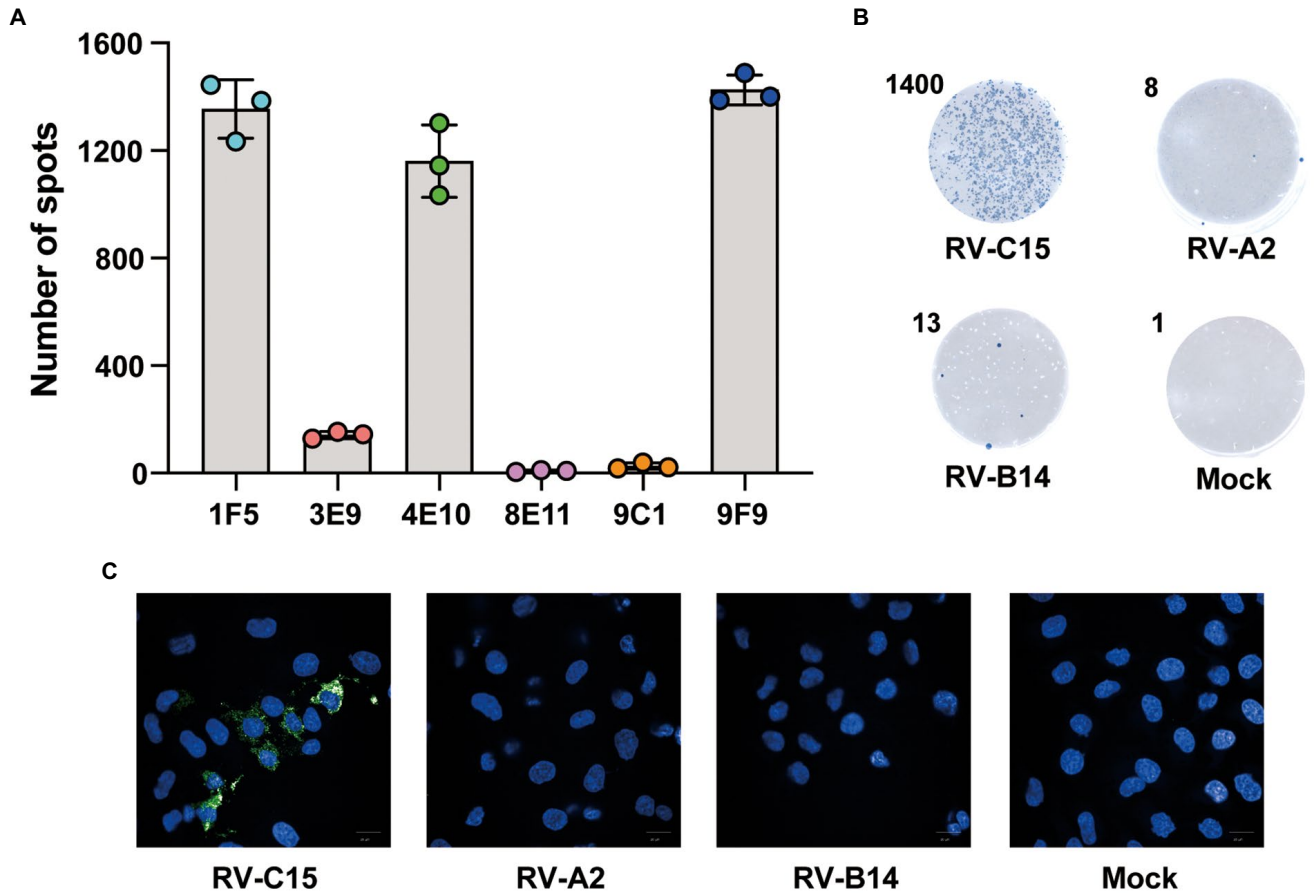
Characterization of the detection antibody in the Nt-ELISPOT. **(A)** Spots detection with different MAbs. Six MAbs produced from mice immunized with RV-C15 were conjugated with HRP and used for the development of the Nt-ELISPOT. Spots were counted by the ImmunoSpot image analyzer. **(B)** Specificity analysis for detection antibody 9F9. RV-C15-, RV-A2-, or RV-B14-infected cells and uninfected cells (Mock) were fixed and incubated with HRP-9F9. The RV-C15-infected cells were visualized as blue spots after staining. Representative cell wells are shown. The number in the upper left corner indicates spots counted by the ImmunoSpot@S5 UV Analyzer. **(C)** Immunofluorescence analysis for detecting antibody 9F9. RV-C15-, RV-A2-, or RV-B14-infected cells and uninfected cells (Mock) were fixed and incubated with 9F9. The secondary antibody was Alexa Fluor^®^ 488 conjugated (green). The nuclei were stained with DAPI (blue). Scale bar = 20 μm.

To obtain MAbs with high reactivity against RV-C15, six MAbs previously produced in our laboratory were selected that could bind to RV-C15-infected cells by ELISPOT assay. Compared with other MAbs, HRP-9F9 showed the highest reactivity to RV-C15 at the same antibody concentration (Figure 3A). As shown in Figure 3B, HRP-9F9 could only react with RV-C15-infected cells, visualized as blue spots, and more than 1,000 blue spots appeared after staining, but did not react with uninfected cells or cells infected with RV-A2 or RV-B14 with a low background. Moreover, immunofluorescence analysis confirmed that 9F9 could only recognize RV-C15-infected cells, but not RV-A2- or RV-B14-infected cells and uninfected cells (Figure 3C). These results indicated that 9F9 specifically recognizes RV-C15 and can be used as a detection antibody in Nt-ELISPOT.

### Determination of the fixative solution for the Nt-ELISPOT

Fixation is considered a key step in the ELISPOT assay. An ideal fixative should preserve both cell morphology and antigenicity of the target protein for antibody detection. Various fixatives, including aldehydes and alcohols, have been used. In this study, 4% paraformaldehyde, 0.2% glutaraldehyde, 1% formaldehyde, 0.5% formaldehyde, or a combination of 1% formaldehyde and 0.1% glutaraldehyde diluted in PBS were used for testing. The results showed that all five fixatives preserved cellular morphology well under the microscope, almost without cellular shedding, but their staining effect was different, which might be due to their influence on antibody–antigen binding (Table 3). Moreover, 1% formaldehyde and 0.5% formaldehyde provided better fixation and staining effect, and the blue spots were clear, uniform, and moderate in size, while the blue spots of other three groups were light in color and unclear (Table 3). Therefore, 0.5% formaldehyde was preferred as the fixative for the Nt-ELISPOT.

**Table 3:** Effect of different fixatives on fixation and staining.

Fixative	Effect on fixation	Effect on staining	RV-C15	Mock
4% Paraformaldehyde	+++	+	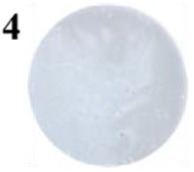	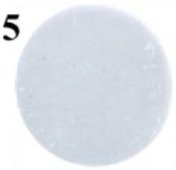
0.2% Glutaraldehyde	+++	+	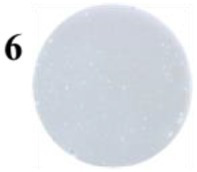	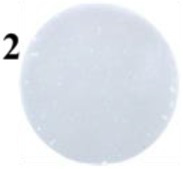
1% Formaldehyde	+++	++++	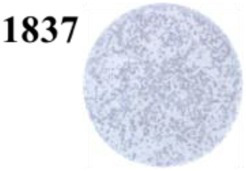	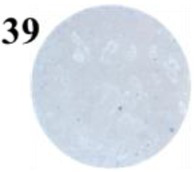
0.5% Formaldehyde	+++	++++	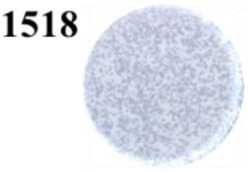	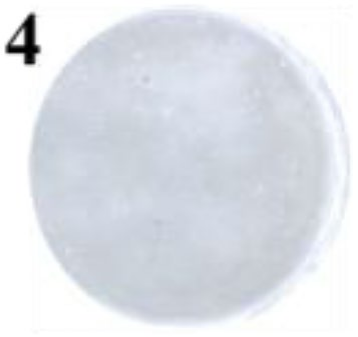
1% Formaldehyde and 0.1% Glutaraldehyde	+++	+	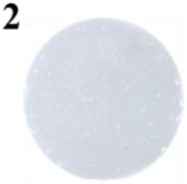	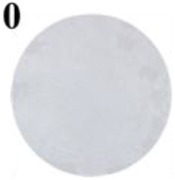

+, effect of fixative on fixation and staining; the more symbols, the better the fixation or staining. Representative ImmunoSpot images using five different fixatives are shown. RV-C15- and mock-infected H1-Hela-R3 cells were counted for comparison.

### Influence of infection parameters for the Nt-ELISPOT

**Figure 4:**
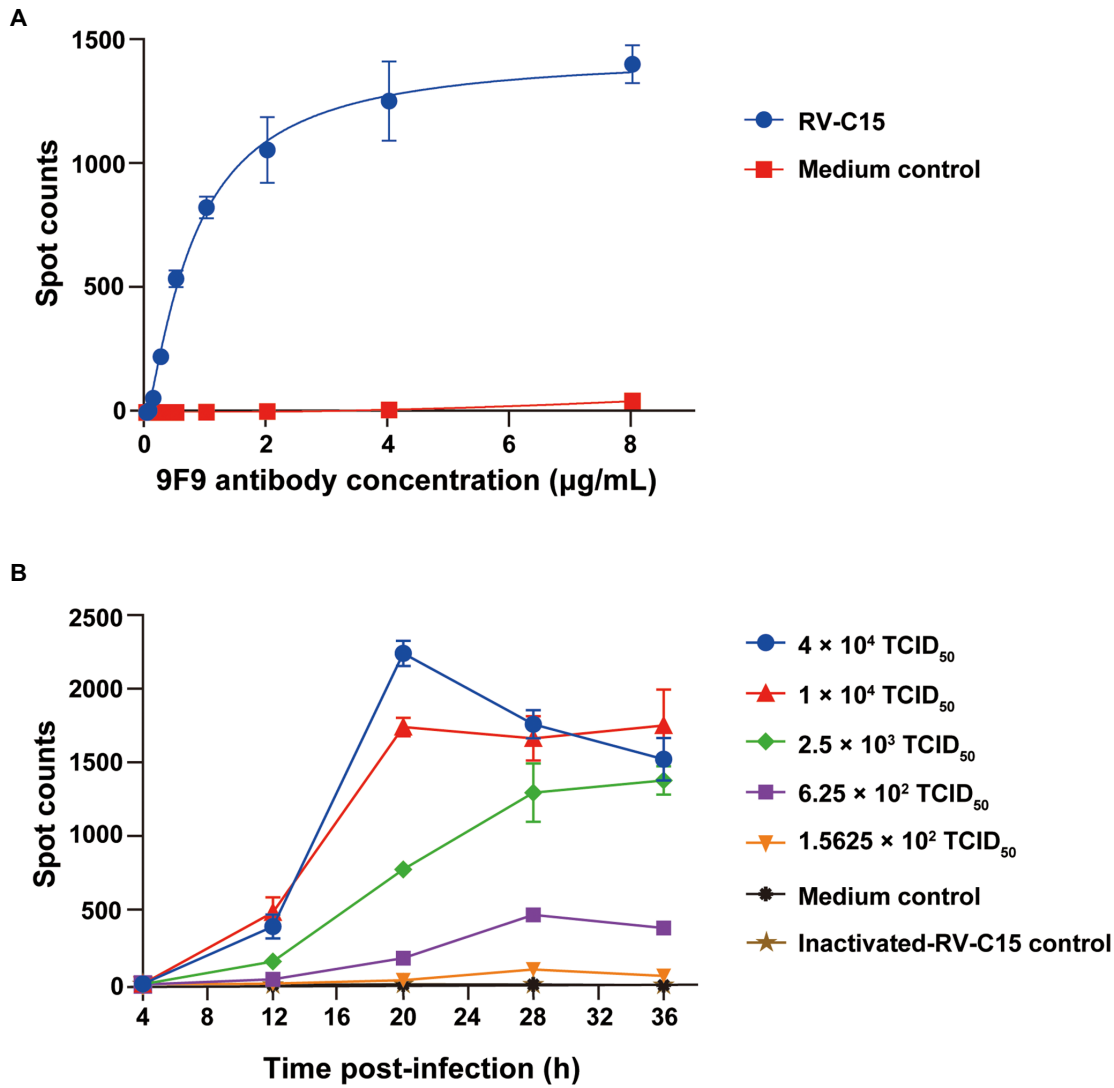
Influence of infection parameters for the Nt-ELISPOT. **(A)** Determination of the optimal antibody concentration for the Nt-ELISPOT. The H1-Hela-R3 cells were infected with RV-C15 at 1 × 10^4^ TCID_50_ per well. The detection was then performed with serially diluted antibodies HRP-9F9. **(B)** Determination of the optimal infectious dose and incubation time for the Nt-ELISPOT. The H1-Hela-R3 cells were infected with RV-C15 in a 4-fold serial dilution from 4 × 10^4^ to 1.5625 × 10^2^ TCID_50_ per well, and the number of spots was counted every 8 h over a period of 36 h using the ELISPOT assay. Inactivated-RV-C15 was inactivated at 56°C for 30 min and then added to the culture wells, which served as a negative control as well as medium group. Each of the dilutions was performed in triplicate. The error bars indicate the mean ± SD from three independent experiments.

Determination of the optimal experimental parameters, including antibody concentration, infectious dose, and incubation time, is a crucial step in the development of 9F9-based Nt-ELISPOT. First, serially 2-fold-diluted HRP-9F9 (initial concentration of 8 μg/ml) was tested for reactivity with RV-C15-infected cells. When the concentration of HRP-9F9 ranged from 2 to 8 μg/ml, the number of spots reached a plateau, with more than 1,000 spots (Figure 4A). Thus, the detection concentration of HRP-9F9 was set at 2 μg/ml. The H1-Hela-R3 cells were infected with RV-C15 in a 4-fold serial dilution from 4 × 10^4^ to 1.5625 × 10^2^ TCID_50_ per well, and the number of spots was counted every 8 h over a period of 36 h. As shown in Figure 4B, spots in the culture wells at a 4 × 10^4^ TCID_50_ dose that could be detected after 12 h, quickly peaked at 20 h post-infection (hpi), and then decreased gradually with the occurrence of obvious viral cytopathic effects. When the infectious dose was 1 × 10^4^ or 2.5 × 10^3^ TCID_50_, spots increased significantly with time and reached a plateau of over 1,000 spots at 20 or 28 hpi. Moreover, spots increased more slowly at lower infectious doses, and the background remained below 50 spots in the uninfected and inactivated-RV-C15-infected cell wells (Figure 4B). Therefore, to improve the detection efficiency and reduce the consumption of the virus, the optimal infectious dose was set at 1 × 10^4^ TCID_50_ when combined with an incubation time of 20 h for the RV-C15 Nt-ELISPOT.

### Comparison of the Nt-CPE and the Nt-ELISPOT

**Figure 5:**
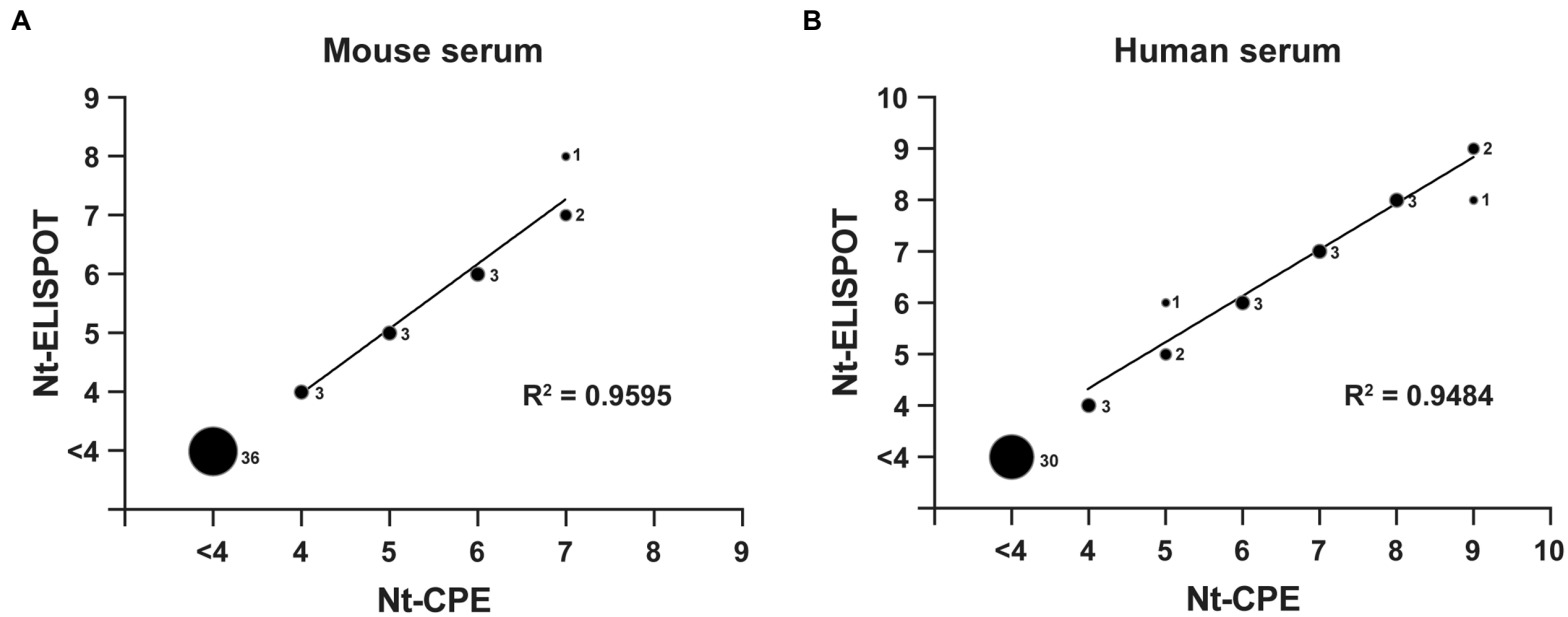
Comparison of the Nt-CPE and the Nt-ELISPOT. A total of 48 mouse serum samples **(A)** and 48 human serum samples **(B)** were serially diluted in serum-free DMEM and tested using both Nt-CPE and Nt-ELISPOT for comparison. The relative size of each spot corresponds to the number of serum samples, as indicated. The neutralization titers were Log2-transformed. Serum with a neutralization titer less than 4 (Log2-transformation) was excluded from the linear regression analyses.

To verify the feasibility of the established RV-C15 Nt-ELISPOT for detecting neutralizing titers in serum samples, the consistency between Nt-ELISPOT and Nt-CPE was evaluated. A total of 12 NAb-positive mouse serum samples, 36 NAb-negative mouse serum samples, 18 NAb-positive human serum samples, and 30 NAb-negative human serum samples were serially diluted in serum-free DMEM and tested using both Nt-CPE and Nt-ELISPOT. As shown in Figure 5, all negative samples were lower than 16, as detected by both Nt-CPE and Nt-ELISPOT, and a good linear relationship was observed between the results of the two methods (*R*^2^ = 0.9896 in Figure 5A, *R*^2^ = 0.9841 in Figure 5B). These data indicated that the results of Nt-ELISPOT were consistent with those of Nt-CPE, and Nt-ELISPOT may be an alternative to Nt-CPE for detecting neutralization titers against RV-C15.

### Screening of anti-RV-C15 neutralizing antibodies using Nt-ELISPOT

**Figure 6:**
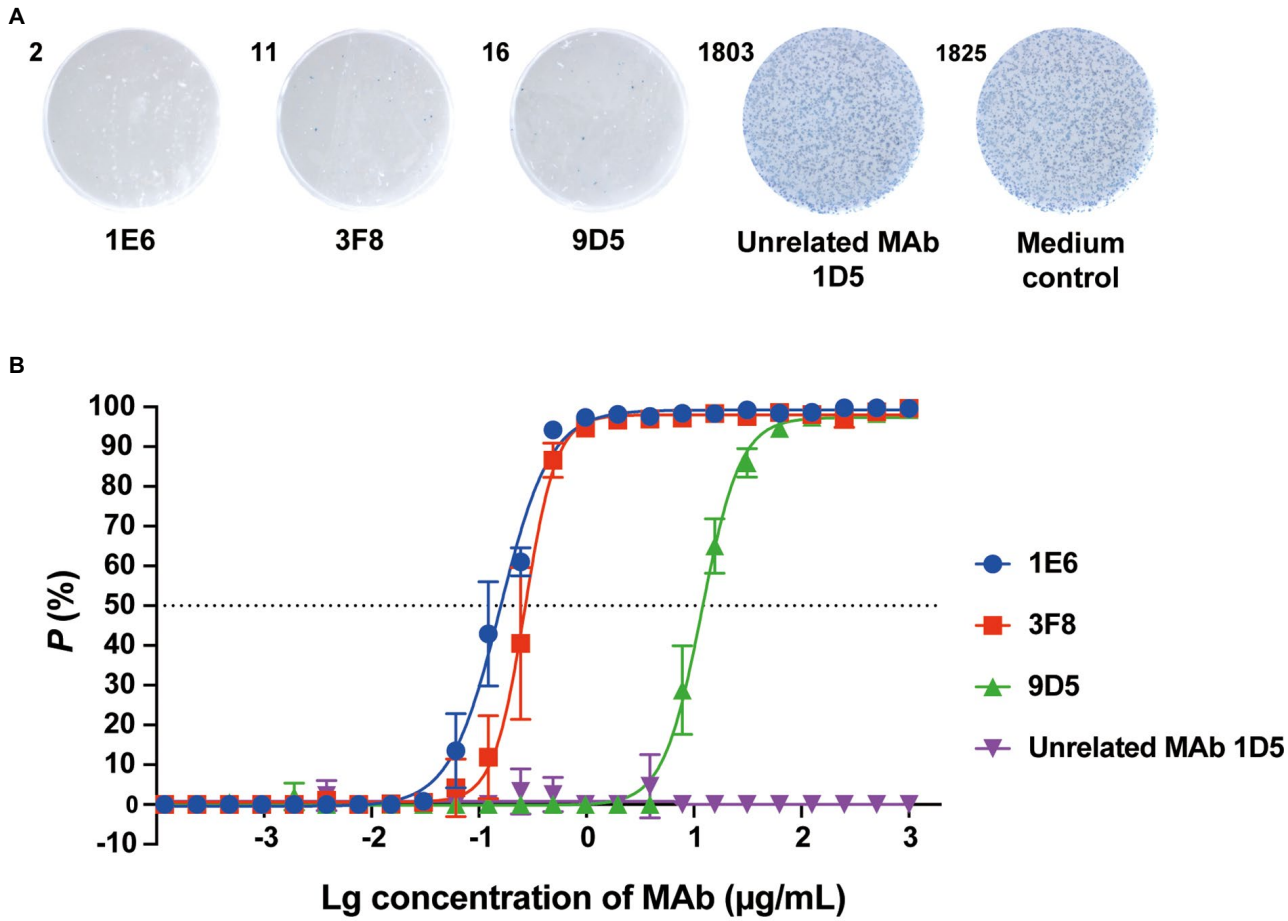
Screening of anti-RV-C15 neutralizing antibodies using Nt-ELISPOT. **(A)** Representative cell wells of anti-RV-C15 neutralizing antibodies, named 1E6, 3F8, and 9D5, screened by Nt-ELISPOT are shown. Unrelated MAb 1D5 (Xu et al., 2017) and medium groups were served as controls. The RV-C15-infected cells were labeled in blue with HRP-9F9. **(B)** The IC_50_ values of the NAbs were calculated by the Nt-ELISPOT. The NAbs 1E6, 3F8, and 9D5 were 2-fold serially diluted for neutralization testing. Unrelated MAb 1D5 was served as a control which showed no significant neutralization efficacy. Each of the dilutions was performed in triplicate. The error bars indicate the mean ± SD from three independent experiments.

Based on the advantages of Nt-ELISPOT, we screened for anti-RV-C15 NAbs using the established Nt-ELISPOT assay. Splenocytes from RV-C15 immunized mice were fused with sp2/0 cells to obtain hybridoma cells, and the hybridoma supernatants were tested by Nt-ELISPOT. After five rounds of testing and cloning, three anti-RV-C15 NAbs were obtained: 1E6, 3F8, and 9D5. As shown in Figure 6A, the NAbs 1E6, 3F8, and 9D5 effectively inhibited the infection of H1-Hela-R3 cells by RV-C15, while cells in the control groups were infected with the virus, and more than 1,000 spots appeared after staining. The neutralizing titers of the three NAbs were determined using Nt-ELISPOT. The IC_50_ values of the NAbs 1E6, 3F8, and 9D5 were 0.16, 0.27, and 11.8 μg/ml, respectively (Figure 6B).

### Detection of serum neutralizing antibodies against RV-C15 using Nt-ELISPOT

**Figure 7:**
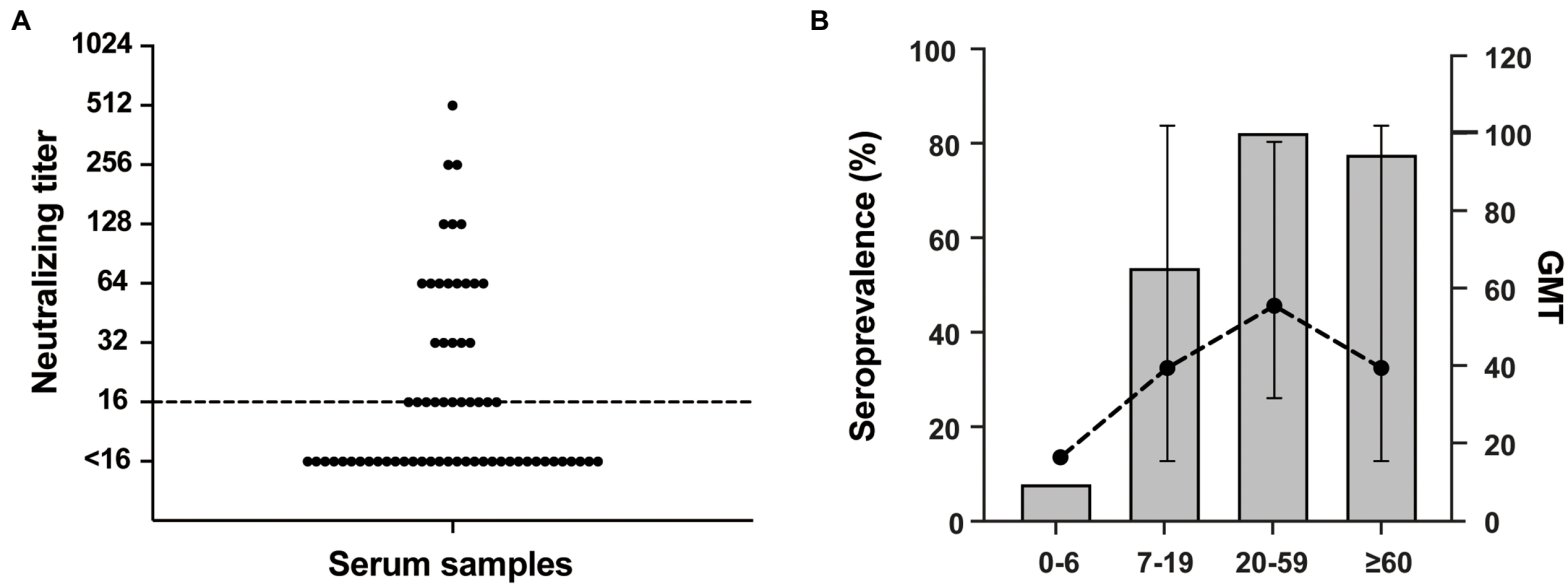
Detection of serum neutralizing antibodies against RV-C15 using Nt-ELISPOT. **(A)** A total of 64 serum samples were collected from a healthy population aged between 6 months and 74 years in Siming District of Xiamen City. The NAb titers against RV-C15 were detected by Nt-ELISPOT. Dotted line indicates the threshold for a positive neutralization titer (≥ 16). **(B)** Among the samples, the seroprevalence of different age groups is shown by histogram. The geometric mean titer (GMT) was calculated as the point plot noted. The bars indicate 95% confidence intervals.

Serological investigations based on NAb detection in the general population are particularly important to assess the prevalence and transmissibility of the virus. The established RV-C15 Nt-ELISPOT was further used to detect serum NAb levels against RV-C15 among a total of 64 serum samples from a healthy population aged between 6 months and 74 years in the Siming District of Xiamen City. As shown in Figure 7A, 31 of 64 serum samples (48.4, 95% CI: 36.6–60.4) were positive for NAbs against RV-C15, and the overall GMT was 43.21 among the positive samples. Moreover, RV-C15 seroprevalence tended to increase with age. The positive rates were 8.0% (2/25, 95% CI: 2.2–25.0), 53.8% (7/13, 95% CI: 29.2–76.8), 82.4% (14/17, 95% CI: 59.0–93.8), and 77.8% (7/9, 95% CI: 45.3–93.7) at the 0–6, 7–19, 20–59 and ≥ 60 age groups, respectively. The GMT of NAb against RV-C15 also showed an increasing trend with age and peaked in the 20–59 age group, with a GMT value of 55.2 (Figure 7B). The results suggested that RV-C15 infection was prevalent in the Siming District of Xiamen City, and children under 6 years of age were more likely to be at high risk for RV-C15 infection.

## Discussion

Nt-ELISPOT, as a highly effective neutralization assay, is considered a powerful tool to develop vaccines and anti-viral drugs against virus infections. The sensitivity and specificity of the Nt-ELISPOT are affected by several parameters, including the cell-fixed solution, infectious dose, and incubation time, in addition to the detection antibody. The ideal fixative should be able to conserve the virus antigenicity. Glutaraldehyde, formaldehyde, and paraformaldehyde are commonly used in cell fixation. After screening, 0.5% formaldehyde in PBS was selected as the cell fixative in the established RV-C15 Nt-ELISPOT because cells were not shed after fixation and staining, and the blue spots were clear and uniform in size. Previous studies have demonstrated that the infectious dose has almost no effect on the neutralizing titers of serum samples, and the infectious dose is negatively correlated with the incubation time within a certain range (Yang et al., 2014). Using a higher infectious dose can further shorten the testing period, but the consumption of virus will also increase. In this study, to improve detection efficiency, the infectious dose was set at 1 × 10^4^ TCID_50_ per well and an incubation time of 20 h was selected as the checkpoint. Additionally, the detection antibody HRP-9F9 can be replaced by other MAbs that bind RV-C15 with high specificity and affinity, such as 1F5 and 4E10 (Figure 3A). The optimal concentration of each detection antibody is different. It is recommended to choose the lowest concentration of antibody when the number of spots reaches the plateau. In this method, each virus-infected cell was visualized as a spot after an enzyme-catalyzed color reaction, which relies on a high-affinity antibody to recognize the specific viral proteins synthesized in cells. Since expression of the viral proteins always occurs before the cytopathic effect, the incubation time of Nt-ELISPOT was greatly shortened compared to that of Nt-CPE.

In recent years, RV-C has become the predominant circulating species, together with RV-A, swapping dominance throughout the years, and it has been found to be more frequently associated with severe disease. Among 57 RV-C subtypes, 13 subtypes, including RV-C2, -C11, -C6, and -C15, were found to be among the subtypes most often detected in clinical studies (Esneau et al., 2022). Surveillance of the seroprevalence and serum NAb levels in susceptible populations is important for understanding previous infection and history of herd immunity, but NAb responses to RV-C have been poorly analyzed. In this study, we preliminarily analyzed NAb levels against RV-C15 in a healthy population in the Siming District of Xiamen City using the established Nt-ELISPOT. The results showed that 48.44% of people were positive for NAb against RV-C15 and the seroprevalence tended to increase with age, suggesting that a high percentage of population had been exposed to RV-C15. The seroprevalence and GMT against RV-C15 were the lowest in the group under 6 years of age, suggesting that children are a high-risk group for RV-C15 infection. However, a relatively small number of samples were tested in this study. Moreover, a COAST (Origin of Children Asthma) longitudinal birth cohort study of nasal and plasma samples, using the real-time PCR, partial sequencing, and a novel neutralization assay based on quantitative PCR, revealed that similar rates of RV-C infection in young children compared with RV-A but proportionally higher rates of RV-C illnesses, and RV-C15 was one of the commonly detected RV-C types (Choi et al., 2021). Thus, to support the development of vaccines and antiviral drugs against RVs, a more complete understanding of the seroepidemiologic characteristics of RVs is necessary. In addition, anti-RV-C NAb has not yet been described. In this study, we obtained three highly efficient NAbs against RV-C15 using the established Nt-ELISPOT, which may be candidates for therapeutic agents against RV-C15 infections.

In summary, this study successfully established a rapid and efficient ELISPOT-based neutralization assay against RV-C15, which can be used for seroepidemiological investigations, efficacy evaluations of vaccines or drugs, and screening of NAbs. However, the established Nt-ELISPOT can only specifically detect NAbs against RV-C15, and the diversity of RVs will limit its widespread use of it. Therefore, it is necessary to obtain high-efficient broad-spectrum detection antibodies against multiple RV subtypes and further establish a universal neutralization assay against RVs.

## Data availability statement

The datasets presented in this study can be found in online repositories. The names of the repository/repositories and accession number(s) can be found in the article/Supplementary material.

## Ethics statement

The studies involving human participants and the use of serum samples were approved by the Ethics Committees of the Fujian Provincial CDC and the Research Ethics Review Committee at Xiamen University. Written informed consent to participate in this study was provided by the participants' legal guardian/next of kin. The animal study was reviewed and approved by All animal experimental protocols were approved by the Xiamen University Laboratory Animal Center (XMULAC) and were conducted in accordance with animal ethics guidelines (approval code: XMULAC20160049).

## Author contributions

ZZ, RZ, HY, LX, TC, JZ, and NX contributed to the experimental design and the manuscript preparation. RZ, HY, LX, ZY, and YW contributed to the virus rescue and cell line construction. ZZ, RZ, HY, LX, QH, and DZ contributed to the preparation and *in vitro* characterization of antibody. ZZ, RZ, HY, and HC contributed to the construction of RV-C15 Nt-ELISPOT. ZZ and RZ contributed to the structural data collection and analysis. All authors contributed to the article and approved the submitted version.

## Funding

This study was supported by grants from the National Natural Science Foundation of China (82101918, 82072282, and 82172248). The funders had no role in the study design, data collection and analysis, decision to publish, or manuscript preparation.

## Conflict of interest

The authors declare that the research was conducted in the absence of any commercial or financial relationships that could be construed as a potential conflict of interest.

## Publisher’s note

All claims expressed in this article are solely those of the authors and do not necessarily represent those of their affiliated organizations, or those of the publisher, the editors and the reviewers. Any product that may be evaluated in this article, or claim that may be made by its manufacturer, is not guaranteed or endorsed by the publisher.
