# Supplementary material for: Development of a rapid neutralization testing system for Rhinovirus C15 based on the enzyme-linked immunospot assay
Source: Front Microbiol. 2022 Sep 23;13:983656. doi: 10.3389/fmicb.2022.983656 (PMC9539532; doi:10.3389/fmicb.2022.983656)
Supplement: Supplementary file 1 [file Data_Sheet_1.DOCX]

# MATERIALS AND METHODS

# Cells, viruses, and serum samples

H1-Hela (ATCC# CRL-1958) and HEK293T/17 (ATCC# CRL-11268) cells were grown in Dulbecco’s modified Eagle’s medium (DMEM; Sigma-Aldrich) supplemented with 10% fetal bovine serum (FBS; Thermo Fisher Scientific), 100 μg/mL streptomycin, 100 U/mL penicillin, and 2 mM L-glutamine. It was then incubated at 37 ℃ with 5% CO_2_. The stable H1-Hela cell line expressing CDHR3 was generated as previously described (1), with the following modifications. Briefly, the gene containing the Y529 FLAG-tagged variant of CDHR3 was synthesized and cloned into a lentiviral plasmid (pLV) expressing puromycin resistance. Recombinant lentiviruses were then packaged by co-transfection with pLV-FLAG-CDHR3Y529, psPAX2 (Addgene plasmid #12260), and pMD2.G (Addgene plasmid #12259) into HEK-293T/17 cells using Lipofectamine 3000 transfection reagent (Thermo Fisher Scientific). The supernatants were harvested 48 h after transfection and then filtered through a 0.45 μm pore size filter. After infection of H1-Hela cells with recombinant lentiviruses and incubation for genome integration, stably transformed cells were selected with puromycin (1 μg/mL) and cloned. The final transduced H1-Hela cell line expressing Y529 FLAG-tagged CDHR3 variants was designated as H1-Hela-R3.

The RV-C15 strain W10 (GenBank accession No.GU219984) infectious clone was prepared by transfecting mRNA transcripts synthesized in vitro using a MEGAscript T7 transcription kit (Thermo Fisher Scientific) from linearized full-length cDNA into H1-Hela-R3 cells, as previously described (1, 2). The rescued RV-C15 was propagated in H1-Hela-R3 cells at 34 ℃, and the supernatants were harvested by freeze-thawing and stored in aliquots at -80 ℃. The virus titer was determined by microtitration using H1-Hela-R3 cells and expressed as the 50% tissue culture infectious dose (TCID_50_), which was calculated by the Reed-Muench method (3). The virus was inactivated at 56 ℃ for 30 min prior to mouse immunization.

NAb levels against RV-C15 in the serum samples were determined using Nt-CPE. A total of 48 mouse serum samples, including 12 NAb-positive mouse serum samples from RV-C15-immunized mice and 36 NAb-negative mouse serum samples from non-immunized mice, were used to evaluate consistency between Nt-ELISPOT and Nt-CPE. A total of 48 human serum samples, including 18 NAb-positive human serum samples and 30 NAb-negative human serum samples, provided by the Center for Disease Control and Prevention (CDC) of Xiamen City, Fujian, China, were used to evaluate the consistency between the two methods. A total of 64 healthy subjects were recruited randomly in the Siming District of Xiamen City in 2016. The 64 serum samples aged between 6 months and 74 years were divided into four age groups, namely, 25 in the 0-6 age group, 13 in the 7-19 age group, 17 in the 20-59 age group, and 9 in the ≥60 years age group. None of the participants showed signs of disease at the time of sample collection. All serum samples were stored at -20 ℃ and inactivated at 56 ℃ for 30 min prior to testing. The use of serum samples was approved by the Ethics Committees of the Fujian Provincial CDC and the Research Ethics Review Committee at Xiamen University.

## Monoclonal antibody production

Five 6-week-old female BALB/c mice (Shanghai Slac Laboratory Animal Co., Ltd., China) were immunized intraperitoneally with RV-C15 (1 × 10^4.5^ TCID_50_/mouse), which was mixed with an equal amount of aluminum adjuvant at 4 ℃ overnight and boosted twice at an interval of 2-week. MAbs were prepared using standard hybridoma technology as previously reported (4). Positive hybridoma cell lines were further generated and screened using indirect ELISA. MAbs were obtained from mouse ascites and purified using protein A chromatography (Bestchrom), according to the manufacturer's instructions. The detection MAbs used in the experiments were conjugated to horseradish peroxidase (HRP) (5) and stored at -20 ℃. All animal experimental protocols were approved by the Xiamen University Laboratory Animal Center (XMULAC) and were conducted in accordance with animal ethics guidelines (approval code: XMULAC20160049).

## Indirect ELISA

The filtered culture supernatants of RV-C15 were diluted in PBS and coated onto 96-well ELISA plates at 4 ℃ overnight. After washing with PBST (0.05% Tween 20 in PBS), the plates were blocked with ED buffer (2% gelatin and 5% casein in PBS) at 37 ℃ for 2 h. Then, the hybridoma cells supernatants were added and incubated at 37 ℃ for 1 h. After five washes with PBST, each well was then incubated with HRP-conjugated goat anti-mouse (HRP-GAM) secondary antibody at a dilution of 1:5000 in ED buffer at 37 ℃ for 30 min. After washing five times with PBST, 100 μL of TMB substrate was added to each well for 10 min to enable development of color before stopping the reaction with 50 μL of 2 M H_2_SO_4_. The absorbance in each well was measured at 450 nm and the background absorbance at 630 nm was subtracted. Positive wells were defined as OD values > 0.1, and negative wells were defined as OD values ≤ 0.1. All tests were performed in triplicates.

## Immunofluorescence Assay

H1-Hela-R3 cells were seeded at 5 × 10^3^ cells/well in 96-well plates and infected with different viruses. Sixteen hours post infection, cells were fixed with 4% paraformaldehyde for 30 min in dark, followed by 0.3% Triton X-100 in PBS for 15 min, and were then blocked with 2% bovine serum albumin (BSA) in PBS at 37 ℃ for 30 min. After washing with PBS, cells were then incubated with MAb 9F9 (initial concentration of 1 mg/mL, at a dilution of 1:1000 in PBS) at 37 °C for 1 h. After three washes with PBST, cells were incubated with GAM-488 (goat anti-mouse IgG labeled with Alexa Fluor® 488, Invitrogen) at a dilution of 1:1000 in PBS at 37 °C for 30 min. After three washes with PBST, the cells were stained with 4', 6-diamidino-2-phenylindole (DAPI, Thermo Fisher Scientific) for 5 min. Images were captured using an Opera Phenix confocal microscope (Perkin Elmer).

## Nt-CPE

H1-Hela-R3 cells were seeded at a density of 5 × 10^3^ cells/well in 96-well plates. Serially 2-fold-diluted serum samples or antibodies (initial concentration, 1 mg/mL) were incubated with an equal volume of RV-C15 (100 TCID_50_) at 34 ℃ for 1 h. The mixtures were then added to the pre-seeded cell plates and incubated for 5 days at 34 °C with 5% CO_2_. Each plate included a cell control and a virus control. The cytopathic effect (CPE) in each well was observed under a microscope and recorded after the incubation. The neutralizing titers were defined as the reciprocal of the highest dilution of cells protected from more than 50% CPE, taken as the average of triplicate data points.

## Statistics

The 50% inhibitory concentration (IC_50_) of MAbs was calculated by nonlinear dose-response regression analysis. The seroprevalence and geometric mean titer (GMT) were calculated. The consistency between Nt-CPE and Nt-ELISPOT results was analyzed using linear regression. The neutralizing titer of sera with values ≥ 16 was considered the threshold for positivity (6-8). Statistical analyses were performed using GraphPad Prism version 8.0. The 95% confidence intervals (CI) for seroprevalence were calculated according to Wilson’s method, using the VassarStats website for statistical computation (<http://vassarstats.net/index.html>).

**REFERENCES**

1. Bochkov YA, Watters K, Ashraf S, Griggs TF, Devries MK, Jackson DJ, et al. Cadherin-related family member 3, a childhood asthma susceptibility gene product, mediates rhinovirus C binding and replication. Proc Natl Acad Sci U S A. 2015;112(17):5485-90.

2. Bochkov YA, Palmenberg AC, Lee WM, Rathe JA, Amineva SP, Sun X, et al. Molecular modeling, organ culture and reverse genetics for a newly identified human rhinovirus C. Nature medicine. 2011;17(5):627-32.

3. REED LJ, MUENCH H. A SIMPLE METHOD OF ESTIMATING FIFTY PER CENT ENDPOINTS12. American Journal of Epidemiology. 1938;27(3):493-7.

4. Varecková E, Betáková T, Mucha V, Soláriková L, Kostolanský F, Waris M, et al. Preparation of monoclonal antibodies for the diagnosis of influenza A infection using different immunization protocols. Journal of immunological methods. 1995;180(1):107-16.

5. Nakane PK, Kawaoi A. Peroxidase-labeled antibody. A new method of conjugation. The journal of histochemistry and cytochemistry : official journal of the Histochemistry Society. 1974;22(12):1084-91.

6. Liu D, Xu L, Zhu R, Yin Z, Lin Y, Hou W, et al. Development of an efficient neutralization assay for Coxsackievirus A10. Appl Microbiol Biotechnol. 2019;103(4):1931-8.

7. Liu CC, Guo MS, Lin FH, Hsiao KN, Chang KH, Chou AH, et al. Purification and characterization of enterovirus 71 viral particles produced from vero cells grown in a serum-free microcarrier bioreactor system. PLoS One. 2011;6(5):e20005.

8. Zhu R, Cheng T, Yin Z, Liu D, Xu L, Li Y, et al. Serological survey of neutralizing antibodies to eight major enteroviruses among healthy population. Emerg Microbes Infect. 2018;7(1):2.
